# Acute renal effects of the GLP-1 receptor agonist exenatide in overweight type 2 diabetes patients: a randomised, double-blind, placebo-controlled trial

**DOI:** 10.1007/s00125-016-3938-z

**Published:** 2016-04-01

**Authors:** Lennart Tonneijck, Mark M. Smits, Marcel H. A. Muskiet, Trynke Hoekstra, Mark H. H. Kramer, A. H. Jan Danser, Michaela Diamant, Jaap A. Joles, Daniël H. van Raalte

**Affiliations:** Department of Internal Medicine/Diabetes Center, VU University Medical Center Amsterdam, De Boelelaan 1117, 1081 HV Amsterdam, the Netherlands; Department of Health Sciences and the EMGO Institute for Health and Care Research, VU University Amsterdam, Amsterdam, the Netherlands; Department of Epidemiology and Biostatistics, VU University Medical Center, Amsterdam, the Netherlands; Division of Pharmacology and Vascular Medicine, Department of Internal Medicine, Erasmus University Medical Center, Rotterdam, the Netherlands; Department of Nephrology and Hypertension, University Medical Center, Utrecht, the Netherlands

**Keywords:** Diabetes, Exenatide, Glomerular filtration rate, Glomerular hyperfiltration, GLP-1 receptor agonist, Glucagon-like peptide-1, Renal function, Renal haemodynamics, Type 2 diabetes

## Abstract

**Aims/hypothesis:**

This study aimed to investigate the acute renal effects of the glucagon-like peptide-1 receptor agonist (GLP-1RA) exenatide in type 2 diabetes patients.

**Methods:**

We included overweight (BMI 25–40 kg/m^2^) men and postmenopausal women, aged 35–75 years with type 2 diabetes (HbA_1c_ 48–75 mmol/mol; 6.5–9.0%) and estimated GFR ≥ 60 ml min^−1^ 1.73 m^−2^. Exenatide or placebo (NaCl solution, 154 mmol/l) was administrated intravenously in an acute, randomised, double-blind, placebo-controlled trial conducted at the Diabetes Center VU University Medical Center (VUMC). GFR (primary endpoint) and effective renal plasma flow (ERPF) were determined by inulin and para-aminohippurate clearance, respectively, based on timed urine sampling. Filtration fraction (FF) and effective renal vascular resistance (ERVR) were calculated, and glomerular hydrostatic pressure (P_GLO_) and vascular resistance of the afferent (R_A_) and efferent (R_E_) renal arteriole were estimated. Tubular function was assessed by absolute and fractional excretion of sodium (FE_Na_), potassium (FE_K_) and urea (FE_U_), in addition to urine osmolality, pH and free water clearance. Renal damage markers, BP and plasma glucose were also determined.

**Results:**

Of the 57 patients randomised by computer, 52 were included in the final analyses. Exenatide (*n* = 24) did not affect GFR (mean difference +2 ± 3 ml min^−1^ 1.73 m^−2^, *p* = 0.489), ERPF, FF, ERVR or P_GLO_, compared with placebo (*n* = 28). Exenatide increased R_A_ (*p* < 0.05), but did not change R_E_. Exenatide increased FE_Na_, FE_K_, urine osmolality and pH, while FE_U_, urinary flow and free water clearance were decreased (all *p* < 0.05). Osmolar clearance and renal damage makers were not affected. Diastolic BP and mean arterial pressure increased by 3 ± 1 and 6 ± 2 mmHg, respectively, whereas plasma glucose decreased by 1.4 ± 0.1 mmol/l (all *p* < 0.05).

**Conclusions/interpretation:**

Exenatide infusion does not acutely affect renal haemodynamics in overweight type 2 diabetes patients at normal filtration levels. Furthermore, acute GLP-1RA administration increases proximal sodium excretion in these patients.

***Trial registration*:**

ClincialTrials.gov NCT01744236

***Funding*:**

The research leading to these results has been funded from: (1) the European Community’s Seventh Framework Programme (FP7/2007-2013) under grant agreement number 282521 – the SAFEGUARD project; and (2) the Dutch Kidney Foundation, under grant agreement IP12.87.

**Electronic supplementary material:**

The online version of this article (doi:10.1007/s00125-016-3938-z) contains peer-reviewed but unedited supplementary material, which is available to authorised users.

## Introduction

Diabetic kidney disease (DKD) has become the leading cause of chronic and end-stage renal disease worldwide. Despite current multifactorial treatment approaches to halt the development and progression of DKD in type 2 diabetes, residual renal risk remains high [1]. Although all glucose-lowering agents reduce hyperglycaemia-associated renal risk, mounting evidence suggests that certain agents may alter risk factors for DKD ‘beyond glycaemic control’ [1].

Glucagon-like peptide (GLP)-1 receptor agonists (GLP-1RAs), which are glucose-lowering agents based on the gut-derived incretin-hormone GLP-1, are now widely used in the management of type 2 diabetes. GLP-1RAs improve glycaemia by stimulating insulin and suppressing glucagon secretion in a glucose-dependent manner [2], but have also been associated with several extra-pancreatic actions, including effects on the kidney [1, 3]. Shortly after Food and Drug Administration (FDA) approval of the first GLP-1RA (exenatide) in the USA in 2005, sporadic case reports described the occurrence of acute renal failure following treatment initiation in type 2 diabetes patients. However, to date, such associations have not been supported by large database analyses or (ongoing) clinical trials [4]. In contrast, more recent evidence suggests that GLP-1RAs may exhibit renoprotective properties beyond glucose lowering. As such, GLP-1RAs reduce albuminuria, a surrogate renal endpoint, in numerous phase III clinical trials [1, 3], and albuminuria progression was reduced in the cardiovascular safety outcome study of the GLP-1RA lixisenatide in patients with type 2 diabetes [5].

Several mechanisms by which GLP-1RAs may affect renal outcome have been proposed. First, GLP-1RAs lower systolic BP by ∼2 mmHg and body weight by ∼3 kg during long-term treatment, which may reduce renal complications [1]. In addition, direct protective effects on the kidney could be involved. As such, GLP-1RAs have been suggested to rapidly reduce glomerular hydrostatic pressure (P_GLO_) and (single-nephron) hyperfiltration, the second of these being a known renal risk factor in diabetes [1, 3]. Indeed, infusion of the GLP-1 peptide increased sodium excretion and reduced creatinine clearance in obese hyperfiltrating insulin-resistant men [6], suggestive of a diuretic action at the level of the proximal tubule, leading to activation of tubuloglomerular feedback (TGF) [6, 7]. However, subsequent studies in normofiltrating healthy men reported neutral renal haemodynamic effects following GLP-1-infusion [6, 8, 9], or even increases in GFR, effective renal plasma flow (ERPF) and estimated P_GLO_ after acute exenatide administration [10]. To date, the renal effects of GLP-1RA therapy in patients with type 2 diabetes remain unknown.

The current study aimed to assess the effects of the GLP-1RA exenatide on gold-standard-measured renal haemodynamics, as well as tubular function and renal damage markers, in overweight patients with type 2 diabetes. We hypothesised that GLP-1RAs reduce GFR and P_GLO_ in type 2 diabetes acutely, i.e. independent of chronic changes in body weight or composition, by stimulating TGF.

## Methods

### Trial design

This was an acute, randomised, double-blind, placebo-controlled trial designed to assess the acute effects of the GLP-1RA exenatide on renal physiology in patients with type 2 diabetes, as described previously [11]. The study was approved by the ethics review board of the VU University Medical Center (VUMC) and local authorities. The study was registered at ClinicalTrials.gov (NCT01744236) and conducted in accordance with the Declaration of Helsinki and the International Conference on Harmonization of Good Clinical Practice. All patients provided written informed consent before participation.

### Study population

The inclusion and exclusion criteria of the study have been reported previously [11]. In brief, white, overweight (BMI 25–40 kg/m^2^) men and postmenopausal women, aged 35–75 years, with type 2 diabetes (HbA_1c_ 48–75 mmol/mol; 6.5–9.0%) were recruited by advertisements in local newspapers. Patients were on a stable dose of metformin and/or sulfonylurea for at least 3 months prior to inclusion. Exclusion criteria included: use of diuretics that could not be stopped for the duration of the study; a history of pancreatic disease; active liver disease; malignancy; estimated GFR < 60 ml min^−1^ 1.73 m^−2^; current urinary tract infection or active nephritis; and neurogenic bladder (an ultrasonic bladder scan was performed to ensure total bladder emptying).

### Intervention and randomisation

Patients were randomised by the trial pharmacist to receive either exenatide or placebo, with an allocation ratio of 1:1 and a block size of six, using computer-generated lists. The pharmacist provided a randomisation list to an independent study physician, who prepared and administered the study drugs but was not involved in data collection or analyses. Because exenatide–placebo pens for subcutaneous administration were not available, the study drug was administered intravenously, thereby allowing blinding of both participants and study personnel. In addition, such administration allows for more stable exenatide plasma levels. The intravenous solution contained 46 ml of NaCl solution (154 mmol/l) and 4 ml of the participant’s blood (to prevent binding of the study drug to the infusion material), with either 10 μg exenatide (AstraZeneca, London, UK) or an equivalent volume of NaCl solution 154 mmol/l (placebo). This schedule has been previously shown to yield plasma exenatide levels within the therapeutic range (130–150 pg/ml), with identical pharmacokinetics as observed after subcutaneous injection, and is well tolerated [12, 13].

### Study protocol

Two days prior to the study visit, participants were instructed to adhere to an average intake of NaCl (9–12 g/day) and protein (1.5–2.0 mg/kg/day) to reduce diet-induced variation in renal physiology. In addition, participants were instructed to refrain from vigorous physical activity and alcohol ingestion for >24 h and from using caffeine or nicotine for >12 h prior to the experiments. After an overnight fast, participants were asked to drink 500 ml of tap water to stimulate diuresis and to delay all medication until conclusion of the experiments, except for their morning dose of metformin.

Participants arrived at the clinical research unit of the Diabetes Center, VUMC, at 07:30 hours. A venous cannula was inserted into an antecubital vein of the dominant arm for infusion of the study drug and renal tracer substances. A second cannula was inserted into an antecubital vein in the contralateral arm for blood sampling. Blood and urine were collected, after which participants assumed a semi-recumbent position in a temperature-controlled room (23.0 ± 1.0°C).

**Fig. 1:**
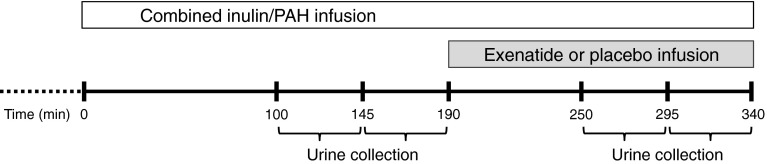
Outline of experimental procedures

After an acclimatisation period of 60 to 90 min [11], infusion of inulin (Inutest, Fresenius Kabi Austria, Graz, Austria) and aminohippurate sodium (PAH; 20%, Merck Sharp & Dohme International, Merck, Whitehouse Station, NJ, USA) was primed with 45 mg/kg and 6 mg/kg body weight, respectively. Thereafter, maintenance infusion was started at 22.5 mg/min for inulin (target plasma concentration 250 mg/l) and 12.7 mg/min for PAH (target plasma concentration 20 mg/l). Following a 90 min equilibration period, urine was collected by spontaneous voiding every 45 min for two periods, which was repeated after 60 min of study-drug infusion (Fig. 1). Diuresis was induced by oral intake of 10 ml/kg (maximum 1000 ml) tap water during the inulin/PAH equilibration period, followed by 200 ml/h of tap water for the remainder of the study.

Participants were allowed to be upright during voiding and encouraged to urinate until a subjective feeling of total bladder emptying was reached. Before and after each urine-collection period, blood samples were taken. Inulin and PAH were measured in all urine and blood samples. Urinary sodium, potassium, urea, osmolality and pH were measured at the second baseline and both acute intervention urine collections, and corresponding blood samples were analysed for electrolytes. The second of two consecutive urine collections was used to measure urinary markers of renal damage, including albumin, neutrophil gelatinase-associated lipocalin (NGAL) and kidney injury molecule-1 (KIM-1), in addition to creatinine and glucose. Haematocrit was determined between two urine-collection periods. Insulin was measured prior to the renal tests, and before the first and after the last urine-collection period of the acute intervention. Plasma renin concentration (PRC) was determined before the renal tests and after the final collection period. Intravenous lines were flushed with 2 ml NaCl solution 154 mmol/l after blood sampling, and a 10 ml/h infusion of NaCl solution 154 mmol/l was sustained throughout the testing day, corresponding to a total volume load of 90 ml and a sodium load of ∼0.8 g during the renal tests. Body water percentage was assessed between the two urine-collection periods, using the single-frequency, bioelectrical impedance analyser Maltron BF-906 (Maltron International, Rayleigh, UK).

### Assays

Venous blood was drawn from the intravenous cannula using syringes, directly transferred to designated BD Vacutainer tubes (Franklin Lakes, NJ, USA) and centrifuged at 1370 *g* for 10 min at 4°C. Fasting plasma glucose, HbA_1c_ (high-performance liquid chromatography) and other baseline laboratory variables were measured before the renal experiments. Venous blood glucose was measured using a YSI-2300 STAT Glucose Analyser (YSI Life Sciences, Yellow Springs, OH, USA) throughout the study, whereas the first plasma glucose and urine glucose were measured using the Gluco-Quant-hexokinase method on a Modular-P (Roche Diagnostics, Basel, Switzerland). Haematocrit was determined using the automated Cell-Dyn Sapphire (Abbott Diagnostics, Abbott Park, IL, USA). Urinary and plasma sodium and potassium were measured using the indirect ion-selective electrode method, whereas urea was determined using enzymatic colorimetric tests on a Modular-P auto analyser. Urinary osmolality was assessed by freezing-point depression with a micro-osmometer (Fiske, Norwood, MA, USA). Urinary pH was determined by hand-held VARIO 2 V00 pH meter and SenTix-V electrode (Wissenschaftlich-TechnischeWerkstätten, Weilheim, Germany). Urinary albumin levels were measured using immunonephelometric techniques. Heparin-plasma and urine samples, stored at −80°C before the assay, were used to assess inulin and PAH by colorimetric assay after preparation with p-dimethylamino-benzaldehyde for inulin [14] and trichloroacetic acid and indole-3-acetic acid for PAH [15]. Urine concentrations of KIM-1 and NGAL were determined by sandwich ELISA according to the manufacturer’s specification (R&D Systems, Minneapolis, MN, USA). The intra- and inter-assay variations of NGAL are 4.1% and 3.1%, respectively, and for KIM-1, the variations are 8.8% and 10.7%, respectively. PRC was measured with a commercial immunoradiometric kit (Renin III; Cisbio, Gif-sur-Yvette, France). Insulin was determined from heparin-plasma using an immunometric assay (ADVIA Centaur-XP Immunoassay System, Siemens Healthcare, Erlangen, Germany). The updated HOMA-IR model, HOMA2-IR, was used to estimate insulin resistance from fasting glucose and insulin (www.dtu.ox.ac.uk/homacalculator).

### Study endpoints

The primary endpoint of this study was exenatide-induced change in GFR compared with placebo [11]. Secondary outcomes included all other (intra-)renal haemodynamic variables, renal handling of sodium, potassium and urea, and renal damage markers. The effects of exenatide on BP and blood glucose were also analysed.

### Sample-size calculation

We calculated that a sample size of 13 patients per group should be sufficient to detect a change of at least 15%, assuming an SD of 8 ml/min, α = 0.05 and power (1 − β) of 80% [11]. However, because the current study was embedded in a long-term, three-armed intervention trial in 60 type 2 diabetes patients [11], a total of 30 patients per group were included in this acute intervention study.

### Calculation of renal physiology and markers of kidney damage

GFR and ERPF were calculated from inulin and PAH clearances, respectively, based on timed urine sampling [16] and averaged from consecutive urine-collection periods. Effective renal blood flow (ERBF) was calculated by dividing ERPF by (1 – haematocrit), filtration fraction (FF) by dividing GFR by ERPF, and effective renal vascular resistance (ERVR) by dividing mean arterial pressure (MAP) by ERBF. Intra-renal haemodynamics (i.e. P_GLO_ and afferent and efferent renal vascular resistance [R_A_ and R_E_, respectively]) were estimated according to the model originally described by Gomez [17] (see electronic supplementary material [ESM]). Absolute electrolyte excretion was calculated by multiplying electrolyte concentrations with urine flow. Fractional electrolyte excretion of sodium (FE_Na_), potassium (FE_K_) and urea (FE_U_) was calculated by using inulin as reference substance. Plasma osmolarity was calculated as 2[Na] + [urea] + [glucose]. Osmol clearance was calculated by urine osmolality × urine flow/plasma osmolarity. Free water clearance was calculated as urine flow − osmol clearance. Renal damage markers were corrected for creatinine and renal haemodynamic variables for body surface area, calculated using the Mosteller formula [18].

### Data management and statistics

Data were double entered into an electronic data management system (OpenClinica LLC, version 3.3, Waltham, MA, USA) and exported to the study database. Before deblinding, urine-collection periods were visually inspected. Baseline urine-collection periods characterised by profound collection errors, defined as an inulin extraction ratio of greater or less than 1 SD of the mean, were discarded from the analyses. Before deblinding, we excluded five patients (all randomised to the exenatide group) from the final analyses because of baseline urine-collection errors.

Multivariable linear regression models were performed in the per protocol population, in which the study endpoint of interest was used as a dependent, and treatment group as an independent variable. We additionally included the corresponding baseline value in the model as an independent variable to correct for potential between-group baseline differences. In cases where variables demonstrated a skewed distribution (as assessed by visual inspection of histograms, Q–Q plots and the Shapiro–Wilk test), log transformation was applied. To assess potential effect modification, subgroup analyses (which were not pre-specified, but in line with our a priori hypothesis) were performed: patients with an estimated GFR of greater or less than 90 ml min^−1^ 1.73 m^−2^, baseline measured GFR, FE_Na_ and urinary albumin/creatinine ratio (ACR) of greater or less than median and use of renin–angiotensin–aldosterone system (RAAS) inhibitors.

Endpoint measurements are reported as mean ± SEM, or, in the case of skewed distribution, median (interquartile range [IQR]). A two-sided *p* < 0.05 was considered statically significant. All analyses were performed using SPSS 22.0 (IBM SPSS, Chicago, IL, USA).

## Results

**Fig. 2:**
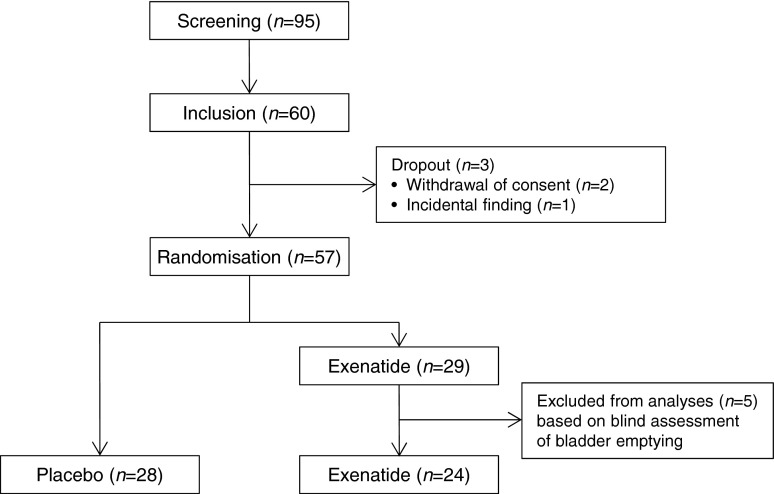
Flow diagram of study participants

Between July 2013 and March 2015, 57 type 2 diabetes patients were randomised (Fig. 2). The final study population comprised 28 patients in the placebo group and 24 patients in the exenatide group. Overall, clinical and biochemistry baseline characteristics were similar between the groups (Table 1).

**Table 1:** Baseline clinical and biochemical characteristics

Characteristic	Placebo (*n* = 28)	Exenatide (*n* = 24)
Clinical characteristics		
Male sex, *n* (%)	23 (82.1)	16 (66.7)
Age, years	64 ± 6	61 ± 8
BMI, kg/m^2^	31.1 (28.2, 34.3)	31.1 (28.4, 33.2)
Current smoker, *n* (%)	6 (21.4)	5 (20.8)
Waist circumference, cm	111.7 ± 8.1	110.6 ± 12.9
Systolic BP, mmHg	136 ± 16	134 ± 13
Diastolic BP, mmHg	76 ± 6	76 ± 7
MAP, mmHg	97 ± 10	96 ± 8
HR, bpm	67 ± 10	64 ± 5
Diabetes history characteristics		
Type 2 diabetes duration, years	7 (4, 13)	7 (4, 10)
Metformin use, *n* (%)	26 (92.9)	23 (95.8)
Sulfonylurea use, *n* (%)	13 (46.4)	10 (41.7)
Antihypertensive medication use, *n* (%)	17 (60.7)	17 (70.8)
RAAS inhibitor use, *n* (%)	16 (57.1)	16 (66.7)
Biochemistry		
HbA_1c_, %	7.3 ± 0.7	7.3 ± 0.6
HbA_1c_, mmol/mol	56 ± 8	57 ± 6
Fasting plasma glucose, mmol/l	8.6 ± 1.9	8.2 ± 1.0
HOMA2-IR	1.81 (1.04, 2.59)	1.56 (1.18, 2.28)
Estimated GFR, ml min^−1^ 1.73 m^−2a^	91 (80, 112)	93 (82, 103)

Data are shown as percentage (%), mean ± SD or median (IQR)

^a^Calculated using the Modification of Diet in Renal Disease (MDRD) study equation: 186 × (serum creatinine [mg/dl])^−1.154^ × (age [year])^−0.203^ × (0.742, if female) [45]

### (Intra-)renal and systemic haemodynamic effects

Exenatide infusion did not affect GFR compared with placebo (mean difference +2 ± 3 ml min^−1^ 1.73 m^−2^, *p* = 0.489) (Table 2; refer to ESM Fig. 1 for individual responses). Also, no between-group differences in ERPF (+3 ± 16 ml min^−1^ 1.73 m^−2^, *p* = 0.852), ERBF (−2 ± 29 ml min^−1^ 1.73 m^−2^, *p* = 0.952) or FF (*p* = 0.166) were observed (Table 2). During exenatide infusion, ERVR tended to increase by 0.018 ± 0.009 mmHg l^−1^ min^−1^ (*p* = 0.065). A significant increase in R_A_ (*p* = 0.010) and no changes in R_E_ or P_GLO_ were observed (Table 2, ESM Fig. 2). Diastolic BP and MAP increased during exenatide vs placebo by 3 ± 1 and 6 ± 2 mmHg (*p* < 0.05), respectively, whereas systolic BP did not change (Table 2). Exenatide infusion increased the heart rate (HR) by 7 ± 1 bpm (*p* < 0.001).

**Table 2:** Haemodynamic, tubular and renal damage marker responses to GLP-1RA exenatide administration in overweight patients with type 2 diabetes

	Placebo (*n* = 28)		Exenatide (*n* = 24)	
Variable	Baseline	Intervention	Baseline	Intervention
Renal haemodynamics				
GFR (ml min^−1^ 1.73 m^−2^)	82 ± 4	83 ± 3	83 ± 3	86 ± 4
ERPF (ml min^−1^ 1.73 m^−2^)	345 ± 18	366 ± 18	343 ± 13	367 ± 18
ERBF (ml min^−1^ 1.73 m^−2^)	605 ± 32	647 ± 35	583 ± 23	623 ± 32
FF	0.244 ± 0.005	0.230 ± 0.003	0.245 ± 0.005	0.236 ± 0.006
ERVR (mmHg l^−1^ min^−1^)	0.184 ± 0.012	0.170 ± 0.010	0.173 ± 0.007	0.176 ± 0.010
P_GLO_ (mmHg)	60 ± 1	60 ± 1	61 ± 1	62 ± 1
R_A_ (dyn s cm^−5^)	6176 ± 607	5712 ± 567	5384 ± 362	6461 ± 670**
R_E_ (dyn s cm^−5^)	3957 ± 105	3732 ± 83	4086 ± 102	3927 ± 109
Renal tubular function				
Na excretion (μmol min^−1^ 1.73 m^−2^)	127 ± 10	153 ± 10	134 ± 10	189 ± 13**
K excretion (μmol min^−1^ 1.73 m^−2^)	62 ± 4	61 ± 5	66 ± 3	65 ± 4
Urea excretion (μmol min^−1^ 1.73 m^−2^)	253 ± 16	243 ± 12	242 ± 14	219 ± 10
FE_Na_ (%)	1.24 ± 0.11	1.33 ± 0.10	1.22 ± 0.10	1.61 ± 0.13***
FE_K_ (%)	21 ± 1	17 ± 1	20 ± 1	20 ± 1*
FE_U_ (%)	70 ± 2	69 ± 1	67 ± 2	64 ± 1**
Urinary pH	5.76 ± 0.11	5.71 ± 0.11	5.91 ± 0.11	6.53 ± 0.12***
Urine osmolality (mOsm/kg)	204 ± 32	225 ± 14	188 ± 15	355 ± 24***
Urinary flow (ml min^−1^ 1.73 m^−2^)	5.2 ± 0.2	3.4 ± 0.1	5.2 ± 0.3	2.4 ± 0.2***
Osmol clearance (ml min^−1^ 1.73 m^−2^)	2.4 (2.0, 2.8)	2.5 (2.3, 2.9)	2.3 (2.1, 2.7)	2.4 (2.1, 2.8)
Free water clearance (ml min^−1^ 1.73 m^−2^)	1.2 ± 3.2	0.9 ± 0.7	1.8 ± 1.7	0.0 ± 0.81***
Renal damage				
ACR (mg/mmol)	1.04 (0.45, 1.80)	0.67 (0.46, 1.04)	0.93 (0.47, 3.21)	0.83 (0.40, 2.22)
NGAL (ng/mmol)	1229 (671, 1890)	1369 (945, 1815)	1460 (798, 3082)	1779 (1068, 2603)
KIM-1 (ng/mmol)	79 (48, 143)	51 (38, 79)	89 (54, 122)	63 (48, 80)
Systemic haemodynamics				
Systolic BP (mmHg)	139 ± 3	145 ± 4	139 ± 2	151 ± 4
Diastolic BP (mmHg)	80 ± 1	80 ± 1	77 ± 1	81 ± 2*
MAP (mmHg)	102 ± 2	103 ± 2	99 ± 2	106 ± 2*
HR (bpm)	67 ± 2	68 ± 2	63 ± 1	70 ± 1***

Data are means ± SEM or median (IQR)

*p < 0.05, **p < 0.01, ***p < 0.001 for exenatide-induced effect vs placebo based on multivariable linear regression, and corrected for potential between-group baseline difference

### Tubular function and renal damage effects

Compared with placebo, exenatide increased absolute sodium excretion by a mean of 34 ± 12 μmol min^−1^ 1.73 m^−2^ (*p* = 0.008), FE_Na_ by 0.30 ± 0.08% (*p* < 0.001) and FE_K_ by 3 ± 1% (*p* = 0.016) (Table 2). Exenatide infusion decreased FE_U_ (3 ± 1%, *p* = 0.002), urinary flow (1.1 ± 0.2 ml min^−1^ 1.73 m^−2^, *p* < 0.001) and free water clearance (0.9 ± 0.2 ml min^−1^ 1.73 m^−2^, *p* < 0.001), while urine osmolality and urinary pH increased (both *p* < 0.001, Table 2). Osmol clearance remained unchanged (*p* = 0.292). Compared with placebo, exenatide did not change urinary glucose, albumin, NGAL or KIM-1 excretion (*p* > 0.05) (Table 2).

### Effects on glucose, body water, renin and exploratory analyses

Blood glucose decreased after exenatide infusion by a time-averaged mean of 1.4 ± 0.1 mmol/l compared with placebo (*p* < 0.001) (ESM Fig. 3), whereas insulin increased by 26 ± 6 pmol/l (*p* < 0.001). Body water percentage did not change throughout the testing day, and no between-group differences were observed (*p* = 0.942). PRC, measured in 12 placebo-treated and nine exenatide-treated patients, did not change in response to exenatide (*p* = 0.401) (ESM Table 1).

Stratification according to baseline-estimated GFR, measured GFR, FE_Na_, ACR or use of RAAS inhibitors (ESM Fig. 4), or correction for between-group differences in glucose or insulin (data not shown) did not change exenatide-induced effects on GFR. In a multiple stepwise regression analysis, exenatide-induced alterations in absolute sodium excretion or FE_Na_ were not explained by alterations in MAP. Exenatide-induced changes in R_A_ were explained by the increase in MAP to a great extent (regression coefficient reduced from 1546 to 794 dyn s cm^−5^, *p* = 0.153) and, in part, by the increase in FE_Na_ (regression coefficient reduced to 1232 dyn s cm^−5^, *p* = 0.047).

### Adverse events

In the exenatide group, four patients experienced nausea without vomiting, while mild headache and diarrhoea occurred in one patient. No adverse events occurred in the placebo group.

## Discussion

The current randomised, placebo-controlled clinical trial is the first to investigate the acute renal effects of a GLP-1RA in overweight patients with type 2 diabetes. We demonstrate that acute intravenous administration of exenatide does not affect gold-standard-measured GFR and ERPF in these patients. In addition, exenatide does not influence FF, P_GLO_ or R_E,_ while it acutely increases R_A_. Absolute sodium excretion, FE_Na_ and FE_K_ increase, while FE_U_, urinary flow and free water clearance decrease. Finally, we demonstrate that exenatide does not affect PRC or urinary markers of renal damage following acute administration.

Several studies have reported effects of GLP-1(RAs) on renal haemodynamics in animals and humans. Previous findings by Gutzwiller et al showed that GLP-1 infusion reduced creatinine clearance measured GFR in 16 obese, hyperfiltrating, insulin-resistant men (three of whom were diagnosed with type 2 diabetes) from 151 ml/min to 142 ml/min [6]. Eight-week treatment with the GLP-1RA exendin-4 also decreased hyperfiltration in a rat model of diabetes [19]. Additionally, an uncontrolled open-label study in 31 normofiltrating patients with type 2 diabetes showed that 7 weeks of treatment with liraglutide decreased GFR and albuminuria, likely by influencing renal haemodynamics [20], effects which were sustained up to 1 year of treatment [21]. Moreover, reductions in albuminuria have been reported in observational studies [20–22]. These studies have led to the hypothesis that GLP-1RAs could confer renoprotection in diabetes by reducing P_GLO_ and glomerular hyperfiltration, in addition to improving the previously mentioned renal risk factors that are affected by long-term GLP-RA treatment, including body weight, BP and albuminuria [1, 3].

However, in the present study, we did not find an acute effect of exenatide infusion on renal haemodynamics in overweight type 2 diabetes patients with normal renal function at baseline. Our findings are in line with some other acute intervention studies in healthy normofiltrating men that examined the effect of intravenous GLP-1 [6, 8, 9]. Remarkably, several preclinical studies reported increases in GFR and ERPF during short-term intervention with GLP-1 [23–25] and GLP-1RAs [7, 26, 27]. It is noteworthy that these studies were performed in animals without diabetes, and GLP-1 and GLP-1RAs were used at doses exceeding human therapeutic concentrations. Moreover, we recently demonstrated that exenatide infusion in ten healthy overweight men increased inulin-measured GFR and PAH-measured ERPF [10]. These contradictory effects of GLP-1(RAs) on renal haemodynamics may be somewhat confusing, but could be due to dissimilar study designs, or could reflect population-dependent effects.

Renal haemodynamics are controlled by intra-renal autoregulatory mechanisms mediated by TGF and myogenic responses, which primarily affect vasomotor tone of the (preglomerular) afferent arteriole [28]. TGF refers to a series of events whereby changes in NaCl concentrations in the tubular fluid are sensed by the cells of the distally located macula densa, eliciting inverse reactions in single-nephron GFR by directly affecting the vascular tone of the afferent arteriole [29]. Furthermore, increased sodium delivery to the macula densa reduces renin secretion. In diabetes, chronic hyperglycaemia leads to augmented renal proximal sodium reabsorption in the proximal tubule, thereby reducing distal NaCl and increasing TGF-regulated (single-nephron) GFR [1, 3]. GLP-1(RAs) have been suggested to restore this maladaptive response by reducing proximal sodium reabsorption [6, 8, 10], and to reduce renin activity [6]. Although exenatide increased sodium excretion in our study, and changes in FE_Na_ partly explained the changes in R_A_, we did not find an effect on GFR or PRC. It could be speculated that GLP-1RAs have little to no TGF-mediated effects in type 2 diabetes patients with normal renal function, or that a potential response is blunted by other effects. Alternatively, although the extensive use of RAAS inhibitors in the current study enabled us to investigate clinically relevant effects on top of standard renoprotective care [1], it may have impeded the possibility of detecting GLP-1RA-induced changes in renal haemodynamics, as these antihypertensive drugs are known to decrease P_GLO_ and (single-nephron) hyperfiltration by reducing R_E_ [3]. However, our analyses did not indicate that these agents modified exenatide-induced effects on GFR.

GLP-1 receptors have been demonstrated in human and monkey smooth muscle cells of the afferent renal arteriole using a validated monoclonal antibody [30]. We previously showed that exenatide reduced R_A_ in overweight men, leading to increases in GFR, ERPF and P_GLO_. This effect was at least partially dependent on NO [10]. These findings are in line with studies in rats, which demonstrated that GLP-1 receptor activation reduces the autoregulatory response of afferent arterioles to an acute increase in pressure [25]. However, we observed an increase in R_A_, which was largely explained by the increase in MAP and, as such, may reflect a (conserved) autoregulatory response activated by stretching of the vascular smooth muscle cells. Although such causality cannot definitely be determined in this study, our findings are compatible with previous studies demonstrating impaired NO-dependent vasodilation in type 2 diabetes patients [31] and vascular resistance to GLP-1RA in swine with metabolic syndrome [32]. Increases in BP following acute GLP-1RA-administration have been widely observed, and both direct sympathetic nervous system activation and reflex tachycardia as a response to vasodilation have been implicated, although the exact mechanism is unclear [33].

The effects of GLP-1(RAs) on tubular electrolyte handling have been reported in several studies. We demonstrate that a GLP-1RA reduces tubular sodium reabsorption and H excretion in type 2 diabetes, in line with previous investigations in healthy and overweight men, and this may help explain the reported GLP-1RA-mediated reductions in BP in clinical trials [6, 8, 10]. Furthermore, we observe an increase in urinary potassium excretion, which indicates that the ratio of sodium reabsorption to potassium secretion is affected in the cortical collecting tubule, as was also observed in preclinical studies [23].

GLP-1(RAs)-mediated natriuresis has been attributed to a reduction in Na^+^/H^+^-exchanger isoform-3 (NHE3) activity in the proximal tubule [6, 8, 10]. Notwithstanding, recent well-performed studies were unable to detect GLP-1 receptors in the tubular lumen [30]. Also, the role of other hormones that influence tubular sodium handling, including angiotensin II [8], are still uncertain, whereas recent studies argue against the involvement of atrial natriuretic peptide [34]. Although exenatide-induced increases in MAP did not explain natriuresis in our analyses, we cannot exclude a role for pressure natriuresis. Interestingly, NHE3 is redistributed and subsequently de-activated in response to an increase in BP [35]. Modest increases in BP have also been observed in other studies reporting GLP-1RA-induced natriuresis [34, 36]. Detailed clinical studies are needed to examine whether renal sodium excretion is sustained after prolonged GLP-1RA treatment.

Exenatide infusion reduced urinary flow, free water clearance and FE_U_ without affecting osmol clearance. These findings are compatible with increases in vasopressin levels or vasopressin receptor activation. Notably, human studies have shown GLP-1 receptor expression in the supraoptic nucleus, which is known to synthesise vasopressin [37]. In rodents, acute GLP-1 administration elevates plasma vasopressin [38], while others reported that vasopressin blockade prevents an acute GLP-1-induced increase in BP [39]. Moreover, a reduction in the gastric emptying rate [2] and/or intestinal sodium absorption [40] may also explain our findings, as this may have led to reduced water absorption. In contrast to our findings, exenatide stimulated free water clearance in rats and humans [41, 42], potentially through prostaglandin E_2_ [43].

Renal damage markers did not change in response to acute exenatide. Although changes were not expected after such short-term drug exposure, the absence of an increase may be important in the light of reported cases of GLP-1RA-associated acute renal failure [3, 4].

Our study has some limitations that need to be addressed. First, we cannot exclude the confounding effects of glucose lowering, which may acutely reduce GFR [44] and could therefore have blunted a potential exenatide-induced increase in GFR (by exenatide per se), or hormonal differences (e.g. insulin), as we did not perform clamp studies. However, the aim of the current study was to assess real-life effects. Second, we excluded five patients from the final analyses, who were, unfortunately, all in the exenatide group. Exclusions were performed in a blinded manner, based on collection errors during baseline measurements, ruling out any influence of exenatide infusion. Importantly, exclusion of these patients, leaving a total of 24 patients in the exenatide group, did not negatively affect our statistical power. Third, the gastric inhibitory effect of exenatide may have reduced gastrointestinal water uptake, thereby reducing urinary flow during testing periods. Fourth, acute renal effects could differ from long-term effects. Finally, estimation of intra-renal haemodynamic variables with the Gomez formulae necessitates assumptions.

In conclusion, we demonstrate that the GLP-1RA exenatide does not affect GFR, ERPF and P_GLO_ in overweight type 2 diabetes patients with normal renal function. In addition, we confirm that GLP-1RA administration increases urinary sodium excretion and urinary pH, which may be due to inhibition of the NHE3 in the proximal tubule or GLP-1RA-induced pressure natriuresis. The long-term renal effects of GLP-1RA in type 2 diabetes remain to be determined.

## Electronic supplementary material

Below is the link to the electronic supplementary material.

ESM Methods (PDF 261 kb)

ESM Fig. 1 (PDF 161 kb)

ESM Fig. 2 (PDF 207 kb)

ESM Fig. 3 (PDF 229 kb)

ESM Fig. 4 (PDF 309 kb)

ESM Table 1 (PDF 222 kb)

## Abbreviations

ACR: Albumin/creatinine ratio
DKD: Diabetic kidney disease
ERBF: Effective renal blood flow
ERPF: Effective renal plasma flow
ERVR: Effective renal vascular resistance
FE_K_: Fractional potassium excretion
FE_Na_: Fractional sodium excretion
FE_U_: Fractional urea excretion
FF: Filtration fraction
GLP-1: Glucagon-like peptide-1
GLP-1RA: Glucagon-like peptide-1 receptor agonist
HR: Heart rate
IQR: Interquartile range
KIM-1: Kidney injury molecule-1
MAP: Mean arterial pressure
NGAL: Neutrophil gelatinase-associated lipocalin
NHE3: Na^+^/H^+^-exchanger isoform-3
PAH: Aminohippurate sodium
P_GLO_: Glomerular hydrostatic pressure
PRC: Plasma renin concentration
R_A_: Afferent renal arteriolar resistance
RAAS: Renin–angiotensin–aldosterone system
R_E_: Efferent renal arteriolar resistance
TGF: Tubuloglomerular feedback
VUMC: VU University Medical Center
