# Supplementary material for: Acute renal effects of the GLP-1 receptor agonist exenatide in overweight type 2 diabetes patients: a randomised, double-blind, placebo-controlled trial
Source: Diabetologia. 2016 Apr 1;59:1412–21. doi: 10.1007/s00125-016-3938-z (PMC4901099; doi:10.1007/s00125-016-3938-z)
Supplement: Supplementary file 1 — (PDF 261 kb) [file 125_2016_3938_MOESM1_ESM.pdf]

ESM accompanying the original article “Acute renal effects of the GLP-1 receptor agonist exenatide in overweight type 2 diabetes patients: a randomised, double-blind, placebo-controlled trial” by Tonneijck L et al

Calculation of intrarenal haemodynamics The filtration pressure across the glomerular capillaries (ΔP_F), is calculated by the following Gomez-formulae [1], assuming the gross filtration coefficient (K_{FG}) to be 0.0551 ml sec^{-1} $mmHg^{-1}$ (given a normal kidney physiology where GFR is 83 ml min^{-1} 1.73 m^{-2}, i.e. mean GFR in the current population), P_{GLO} 60 mmHg (given Winton’s indirect estimates in the dog that glomerular pressure is roughly two-thirds of MAP [2]), and normal glomerular oncotic pressure (π_G) 25 mmHg:

$$\Delta P_F = GFR\ (ml/sec)/\ K_{FG}$$

π_G (mmHg) is calculated from the plasma protein concentration within the glomerular capillaries (C_M). C_M is calculated from the total protein concentration in g/dl (TP) and FF:

$$C_M = TP/FF * Ln(1/1 - FF)$$

$$\pi_G = 5 * (C_M - 2)$$

P_{GLO} is calculated by using variables described above and given the assumption that the hydrostatic pressure in Bowman’s space (P_{BOW}) is 10 mmHg:

$$P_{GLO} = \Delta P_F + P_{BOW} + \pi_G$$

$$P_{GLO} = (GFR/\ K_{FG}) + 10\ mmHg + [5*(TP/FF*Ln(1/ -FF)-2)]$$

In order to calculate R_A and R_E, principles of Ohm’s law are used, and the factor 1328 to convert to dyn s cm^{-5}:

$$R_A = [(MAP - P_{GLO}/RBF]*1328$$

$$R_E = [GFR/(K_{FG}*(RBF - GFR)]*1328$$

References

1. Gomez DM (1951) Evaluation of renal resistances, with special reference to changes in essential hypertension. J Clin Invest 30:1143–1155.
2. Winton F (1937) Physical factors involved in the activities of the mammalian kidney. Physiol Rev 17:408–435.
