# Supplementary material for: Acute renal effects of the GLP-1 receptor agonist exenatide in overweight type 2 diabetes patients: a randomised, double-blind, placebo-controlled trial
Source: Diabetologia. 2016 Apr 1;59:1412–21. doi: 10.1007/s00125-016-3938-z (PMC4901099; doi:10.1007/s00125-016-3938-z)

ESM accompanying the original article "Acute renal effects of the GLP-1 receptor agonist exenatide in overweight type 2 diabetes patients: a randomised, double-blind, placebo-controlled trial" by Tonneijck L et al

ESM Fig. 1 Individual GFR responses to intravenous GLP-1RA exenatide administration in overweight patients with type 2 diabetes

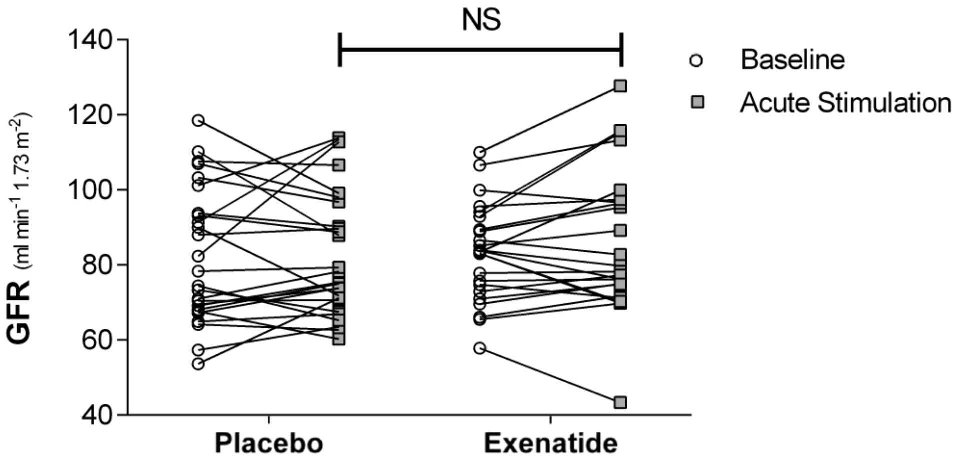

Supplement: Supplementary file 2 — (PDF 161 kb) [file 125_2016_3938_MOESM2_ESM.pdf]
