# Supplementary material for: Acute renal effects of the GLP-1 receptor agonist exenatide in overweight type 2 diabetes patients: a randomised, double-blind, placebo-controlled trial
Source: Diabetologia. 2016 Apr 1;59:1412–21. doi: 10.1007/s00125-016-3938-z (PMC4901099; doi:10.1007/s00125-016-3938-z)
Supplement: Supplementary file 4 — (PDF 229 kb) [file 125_2016_3938_MOESM4_ESM.pdf]

ESM accompanying the original article “Acute renal effects of the GLP-1 receptor agonist exenatide in overweight type 2 diabetes patients: a randomised, double-blind, placebo-controlled trial” by Tonneijck L et al

ESM Fig. 3 Blood glucose response to intravenous GLP-1RA exenatide administration in overweight patients with type 2 diabetes

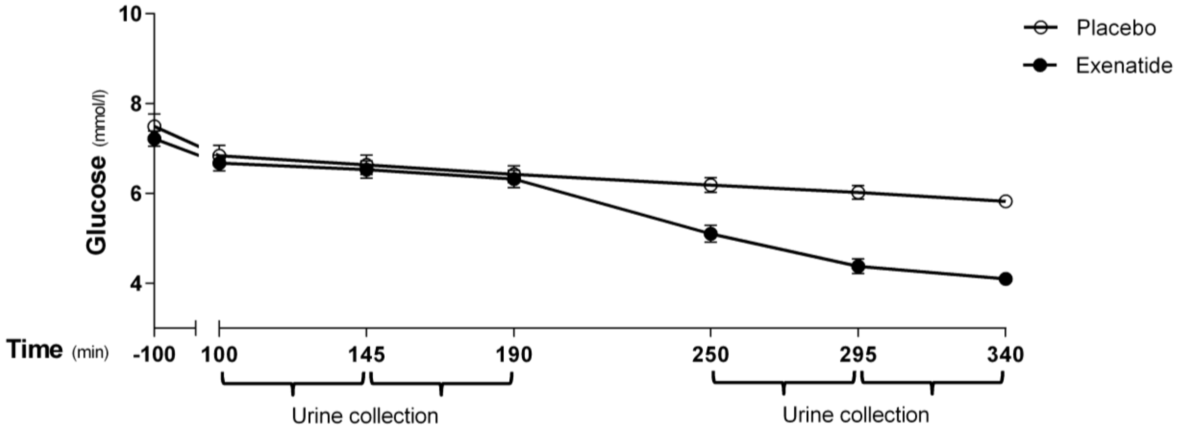

Blood glucose decreased after exenatide infusion by a time-averaged mean of 1.4±0.1 mmol/l compared with placebo ($p<0.001$).
