# Supplementary material for: Acute renal effects of the GLP-1 receptor agonist exenatide in overweight type 2 diabetes patients: a randomised, double-blind, placebo-controlled trial
Source: Diabetologia. 2016 Apr 1;59:1412–21. doi: 10.1007/s00125-016-3938-z (PMC4901099; doi:10.1007/s00125-016-3938-z)
Supplement: Supplementary file 5 — (PDF 309 kb) [file 125_2016_3938_MOESM5_ESM.pdf]

ESM accompanying the original article "Acute renal effects of the GLP-1 receptor agonist exenatide in overweight type 2 diabetes patients: a randomised, double-blind, placebo-controlled trial" by Tonneijck L et al

ESM Fig. 4 Subgroup-analyses of GFR response to intravenous GLP-1RA exenatide administration in overweight patients with type 2 diabetes

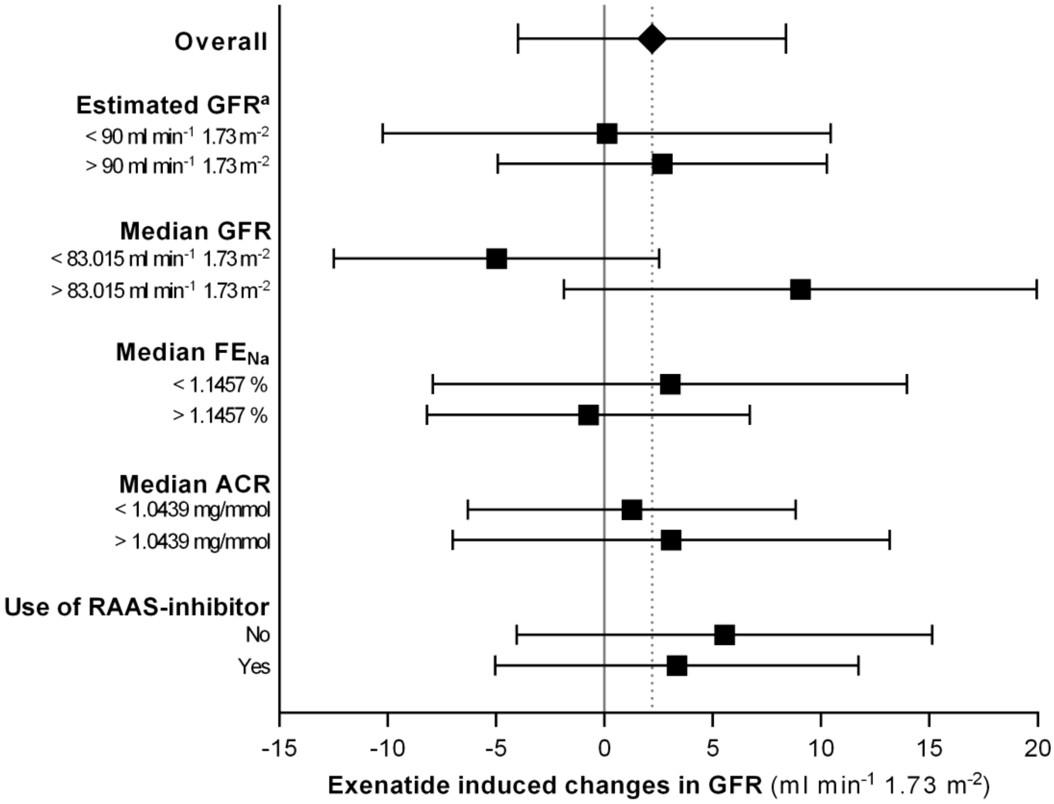

Data are shown as mean (95% confidence interval)

[a]Calculated using the MDRD study equation: 186 × (serum creatinine [mg/dl])$^{-1.154}$ × (age [year])$^{-0.203}$ × (0.742, if female) [1]

References

1. Myers GL, Miller WG, Coresh J, et al. (2006) Recommendations for improving serum creatinine measurement: A report from the Laboratory Working Group of the National Kidney Disease Education Program. Clin Chem 52:5–18.
