# Supplementary material for: Acute renal effects of the GLP-1 receptor agonist exenatide in overweight type 2 diabetes patients: a randomised, double-blind, placebo-controlled trial
Source: Diabetologia. 2016 Apr 1;59:1412–21. doi: 10.1007/s00125-016-3938-z (PMC4901099; doi:10.1007/s00125-016-3938-z)
Supplement: Supplementary file 6 — (PDF 222 kb) [file 125_2016_3938_MOESM6_ESM.pdf]

ESM accompanying the original article "Acute renal effects of the GLP-1 receptor agonist exenatide in overweight type 2 diabetes patients: a randomised, double-blind, placebo-controlled trial" by Tonneijck L et al

ESM Table 1 Baseline characteristics of the subgroup in which renin response to intravenous GLP-1RA exenatide administration in overweight patients with type 2 diabetes was measured

**Characteristic**	**Placebo (n=12)**	**Exenatide (n=9)**
Clinical characteristics		
Male sex, *n* (%)	11 (91.7)	6 (66.7)
Age, years	65±8	65±6
BMI, kg/m^2	31.8 (30.0, 34.6)	31.8 (28.5, 36.1)
Current smoker, *n* (%)	3 (25.0)	1 (11.1)
Waist circumference, cm	112.2±7.6	112.2±14.8
Systolic BP, mmHg	134±13	140±15
Diastolic BP, mmHg	78±6	78±8
MAP, mmHg	96±8	99±11
HR, bpm	66±10	65±6
Diabetes history characteristics		
Type 2 diabetes duration, years	8 (4, 15)	8 (5, 14)
Metformin use, *n* (%)	11 (91.7)	8 (88.9)
Sulfonylurea use, *n* (%)	6 (50.0)	5 (55.6)
Antihypertensive medication use, *n* (%)	9 (75.0)	9 (100.0)
RAAS inhibitor use, *n* (%)	8 (66.7)	8 (88.9)
Biochemistry		
HbA_{1c}, %	7.5±0.6	7.4±0.5
HbA_{1c}, mmol/mol	58±7	58±6
Fasting plasma glucose, mmol/l	8.8±2.2	8.2±0.8
Estimated GFR, ml min^{-1} 1.73 m^{-2}[a]	92 (78, 105)	80 (78, 92)

Data are shown as percentage (%), mean ± SD or median (IQR)
[a]Calculated using the MDRD study equation: 186 × (serum creatinine [mg/dl])$^{-1.154}$ × (age [year])$^{-0.203}$ × (0.742, if female) [1]

During placebo-infusion PRC changed from median (IQR) 12.9 (3.2, 31.5) to 18.7 (3.7, 29.9) pg/ml, whereas this was from 18.0 (10.3, 31.3) to 15.5 (8.9,18.5) pg/ml during exenatide. No between-group difference was observed (p=0.401).

References

1. Myers GL, Miller WG, Coresh J, et al. (2006) Recommendations for improving serum creatinine measurement: A report from the Laboratory Working Group of the National Kidney Disease Education Program. Clin Chem 52:5–18.
